# Unlocking the bacterial contact-dependent antibacterial activity to engineer a biocontrol alliance of two species from natural incompatibility to artificial compatibility

**DOI:** 10.1007/s44154-021-00018-x

**Published:** 2021-12-16

**Authors:** Qianhua Wu, Bozhen Wang, Xi Shen, Danyu Shen, Bingxin Wang, Qinggang Guo, Tao Li, Xiaolong Shao, Guoliang Qian

**Affiliations:** 1 College of Plant Protection, Laboratory of Plant Immunity, Key Laboratory of Integrated Management of Crop Diseases and Pests, Nanjing Agricultural University, No.1 Weigang, Nanjing, Jiangsu 210095 People’s Republic of China; 2 Institute of Plant Protection, Hebei Academy of Agricultural and Forestry Sciences, Integrated Pest Management Center of Hebei Province, Key Laboratory of IPM on Crops in Northern Region of North China, Ministry of Agriculture and Rural Affairs of China, Baoding, 071000 People’s Republic of China; 3 Shanghai Veterinary Research Institute, Chinese Academy of Agricultural Sciences, Shanghai, 200241 People’s Republic of China

**Keywords:** PGPR, Biocontrol, Contact-dependent antibacterial activity, T4SS, Engineering

## Abstract

Plant growth-promoting rhizobacteria (PGPR) contain various biocontrol bacteria with broad-spectrum antimicrobial activity, and their single species has been extensively applied to control crop diseases. The development of complex biocontrol community by mixing two or more PGPR members together is a promising strategy to enlarge the efficacy and scope of biocontrol. However, an effective method to assess the natural compatibility of PGPR members has not yet been established to date. Here, we developed such a tool by using the bacterial contact-dependent antibacterial activity (CDAA) as a probe. We showed that the CDAA events are common in two-species interactions in the four selected representative PGPRs, represented by the incompatible interaction of *Lysobacter enzymogenes* strain OH11 (OH11) and *Lysobacter antibioticus* strain OH13 (OH13). We further showed that the CDAA between OH11 and OH13 is jointly controlled by a contact-dependent killing device, called the type IV secretion system (T4SS). By deleting the respective T4SS synthesis genes, the T4SS in both strains was co-inactivated and this step unlocked  their natural CDAA, resulting in an engineered, compatible mutant alliance that co-displayed antibacterial and antifungal activity. Therefore, this study reveals that releasing bacterial CDAA is effective to rationally engineer the biocontrol community.

The online version contains supplementary material available at 10.1007/s44154-021-00018-x.

## Introduction

Plant growth-promoting rhizosphere bacteria (PGPR) are a group of beneficial plant bacteria with biocontrol ability. They usually colonize the surfaces of plant roots and protect plants from pathogen infections by producing antimicrobial compounds and inducing plant immune responses (Mendes et al. 2013). In agriculture, a high- performance single PGPR species/strain is widely developed as a “green” bio-pesticide to control multiple crop diseases (Bhattacharyya and Jha, 2012). To expand the scope and efficacy of biocontrol, scientists have tried to engineer complex biocontrol communities with synergistic effects by mixing two or more PGPR members (Massart et al. 2015). One of the key points of this rational design is to develop a general method to assess whether these PGPR members are naturally compatible, but, as far as we know, such a tool has been lacking to date.

The key members of the PGPR group comprise species from gram-positive *Bacillus* and gram-negative *Pseudomonas* and *Lysobacter* (Haas and Defago 2005; Lugtenberg and Kamilova 2009; Fira et al. 2018; Lin et al. 2021). These key members not only produce diffusible antimicrobial compounds (a long-range weapon) to destroy fungal cell membrane and cell wall, but also assemble contact-dependent killing devices such as the type VI (T6SS), type VII (T7SS) and type IV (T4SS) secretion systems for injecting lethal effector proteins to antagonize competing microbes (Haas and Defago 2005; Bernal et al., 2017; Fira et al. 2018, Lin et al. 2021). Among them, T6SS is a contact-dependent weapon generally used by the plant-associated proteobacteria (Bernal et al., 2017; Hachani et al. 2016; Galan and Waksman 2018; Hernandez et al. 2020). Upon cell-cell contact, T6SS transports diverse toxic effectors into the prey cell by piercing the cell wall and cell membrane of the prey (Liang et al. 2019; Hernandez et al. 2020). The beneficial plant bacterium *Pseudomonas putida* has been shown to employ its T6SS to combat bacterial phytopathogens, such as *Xanthomonas campestris* (Bernal et al. 2017). *Bacillus* spp. do not possess T6SS, but rather harbor an Esx system, which resembles a T7SS device that mediates contact-dependent killing of competing bacteria (Bottai et al., 2016). VirB/D4 T4SS has recently been shown to be a new contact-dependent bacterial-killing system, which was originally described in two pathogenic species of *Stenotrophomonas maltophilia* and *Xanthomonas citri* (Souza et al. 2015; Bayer-Santos et al. 2019). The effector proteins translocated by this system contain a conserved C-terminal XVIPCD domain that are lethal to their bacterial competitors (Souza et al. 2015; Bayer-Santos et al. 2019).

A recent work in our laboratory showed that a PGPR member, called anti-fungal *Lysobacter enzymogenes* strain OH11 (OH11), can use the bacterial-killing T4SS to combat another PGPR species-the antibacterial *Pseudomonas protegens* strain Pf-5 (Pf-5), and this killing effect requires their cell-cell contacts (Shen et al. 2021). This suggests that the contact-dependent antibacterial activity between OH11 and Pf-5 is not conducive to their natural combination to co-exhibit antifungal and antibacterial activities. Indeed, we found that OH11 kills Pf-5 through cell-cell contact and remarkably reduces the antibacterial activity expressed by Pf-5 (Shen et al. 2021). This finding raises the possibility that we may be able to develop a feasible approach to help rationally design collaborative biocontrol communities by monitoring and unlocking contact-dependent antibacterial activity among the cell-cell interactions of biocontrol agents.

In this study, we first showed that contact-dependent antibacterial activity is common among the four selected representative PGPR members (one *Bacillus* species; two *Lysobacter* members; and one *Pseudomonas* species). To unlock the observed contact-dependent antibacterial activity in selected PGPR members by biotechnology, we selected a representative, incompatible interaction between two *Lysobacter* species– OH11 and *L. antibioticus* strain OH13 (OH13). OH11 produces the heat-stable antifungal factor, HSAF (Yu et al. 2007; Qian et al. 2013). OH13 produces phenazines and p-aminobenzoic acid (pABA), which have antibacterial and antifungal activity (Zhao et al. 2016; Laborda et al., 2019). We genetically inactivated the T4SS in both OH11 and OH13, which indeed unlocked their natural contact-dependent antibacterial activity and enabled us to artificially generate compatible cell-cell interactions. It is expected that this engineered combination of two species will exhibit both contact-independent antibacterial and antifungal activity. Therefore, our research results provide an effective approach for engineering synergistic biocontrol alliance by unlocking the contact-dependent antibacterial activity.

## Results

### Contact-dependent antibacterial activity is common among cell-cell interactions of selected PGPR members

**Fig. 1:**
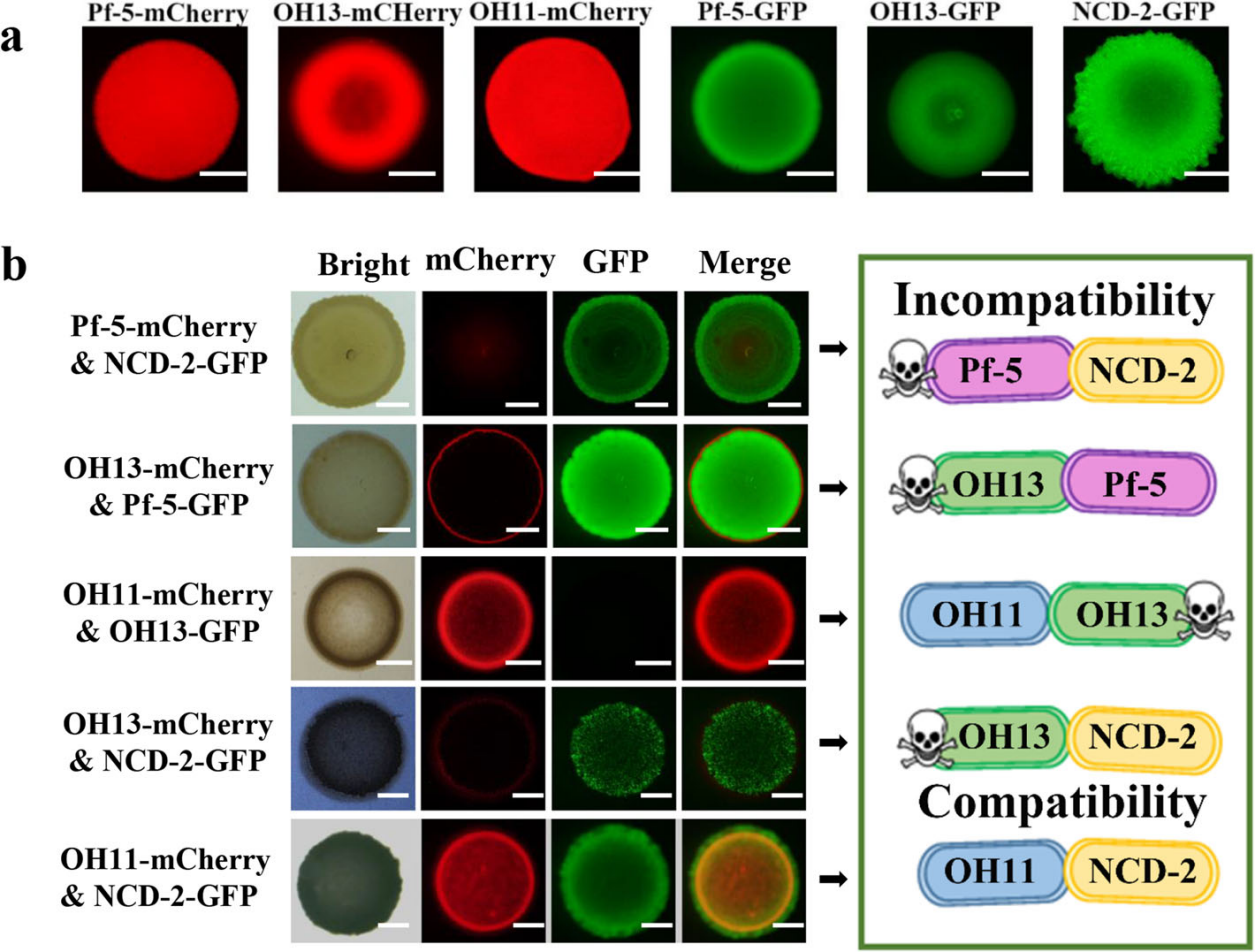
Fluorescence-based assessment of contact-dependent antibacterial activity of interspecies interactions of the four selected PGPR species. (**a**) Fluorescence observation of the single-species cultures of four PGPR species labelled with mCherry or GFP. Pf-5, *Pseudomonas protegens*; OH13, *Lysbacter antibioticus*; OH11, *Lysobacter enzymogenes*; NCD-2, *Bacillus subtilis*. The bar represents 2 mm. (**b**) Evaluation of the contact-dependent antibacterial activity phenomenon by co-cultivating two PGPR species at a ratio of 1:1 on 1/10 TSA agar. The fluorescence signals were observed after 24 h of incubation. On the left, the disappearance of the corresponding fluorescence indicates that one strain labelled with the corresponding fluorescent protein is killed by the other during their co-culturing. On the right, the results of the incompatibility or compatibility of the two-species in the left panel are summarized in cartoons. Skeleton symbols indicate cells that have been killed. The bar represents 2 mm

**Fig. 2:**
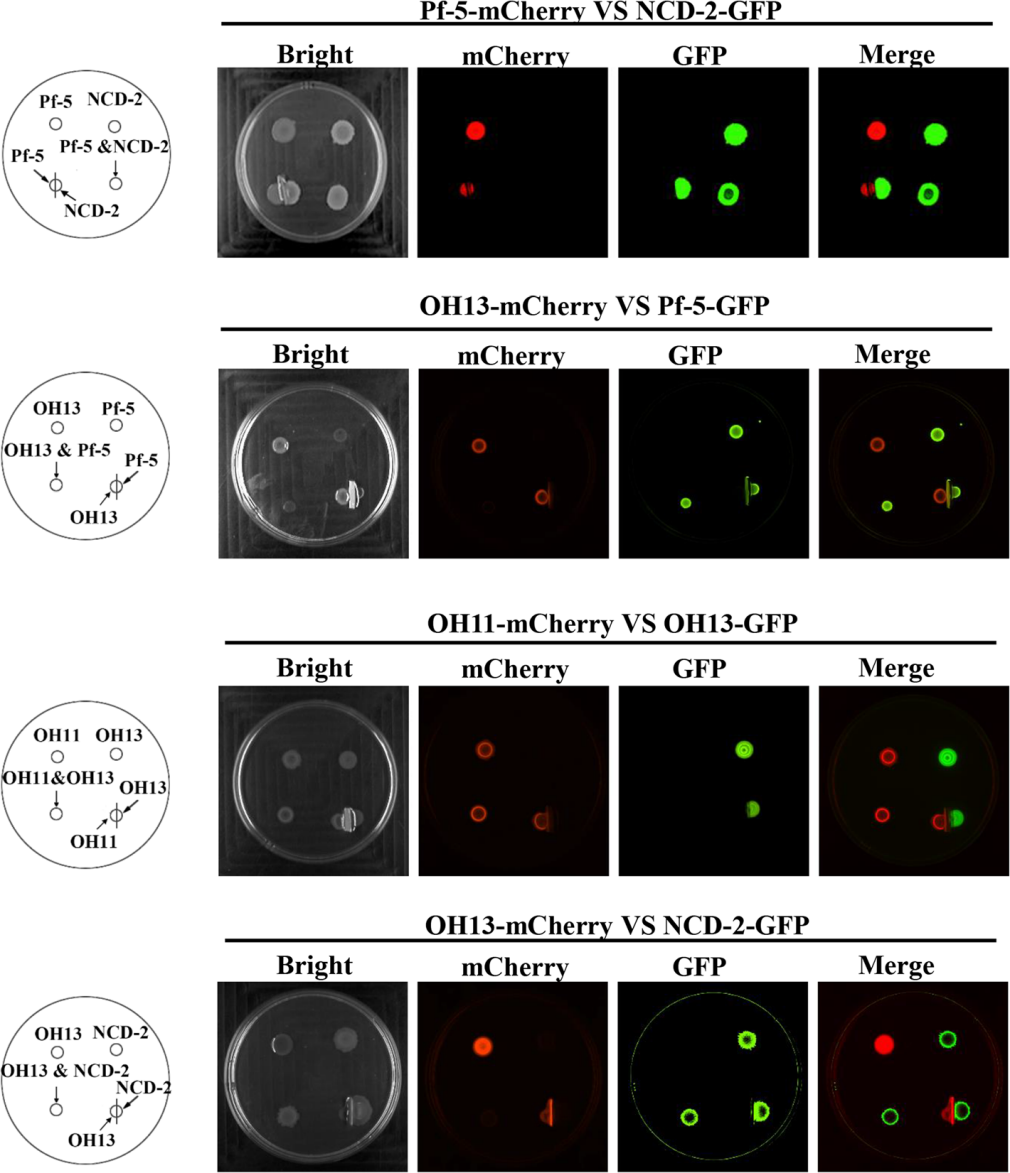
Cell-cell contact is required for contact-dependent inhibition of interspecies interactions of the four selected PGPR species. The cell-cell contact was separated by using a 0.22-μM filter membrane filter. The strains marked with mCherry or GFP are the same shown in Fig. 1b. The fluorescence signals were observed after 24 h of incubation. The bar represents 2 mm

To explore whether contact-dependent antibacterial activity phenomenon is widespread among PGPR members, four representative, well-studied species in biocontrol mechanisms and/or field applications (Table S1): the *Bacillus* NCD-2, the *Lysobacter* OH11 and OH13, and the *Pseudomonas* Pf-5 were selected and genetically labelled by the fluorescent GFP or mCherry (Fig. 1a). Contact-dependent antibacterial activity assays were carried out by randomly designing the following combinations: OH11-OH13, NCD-2-Pf-5, OH13-NCD-2, OH13-Pf-5, and OH11-NCD-2. To create conditions to trigger contact-dependent antibacterial activity in the laboratory, the two- species culture from each combination was mixed at a ratio of 1:1, and further co-cultivated on agar plates. Through this step, we found that except for the OH11-NCD-2 combination, the other four combinations are incompatible, as evidenced by the killing of one of the species via cell-cell contact determined by the fluorescent microscope. In brief, NCD-2 killed Pf-5 and OH13, Pf-5 killed OH13, and OH11 killed OH13 and Pf-5 (Fig. 1b, Shen et al., 2021). Furthermore, the observed contact-dependent antibacterial activity events described in Fig. 1 require cell-cell contact, because separate cultivation of two selected species on the same media by a 0.22-μM membrane filter does not result in a killing effect (Fig. 2). These results together uncover that contact-dependent antibacterial activity can be used as a powerful probe to evaluate the contact-dependent, compatible or incompatible interactions of PGPR species.

### Contact-dependent antibacterial activity locked *L. enzymogenes*-*L. antibioticus* interaction to co-express contact-independent antimicrobial activity

The discovery of contact-dependent antibacterial activity in most of the selected PGPR species indicates that a simple mixing is not a reasonable strategy for engineering a two-species biocontrol alliance. To test this hypothesis, we chose the incompatible OH11-OH13 combination, because in the past ten years, we have established mature systems for these two species (Lin et al., 2021; Xu et al., 2021).

**Fig. 3:**
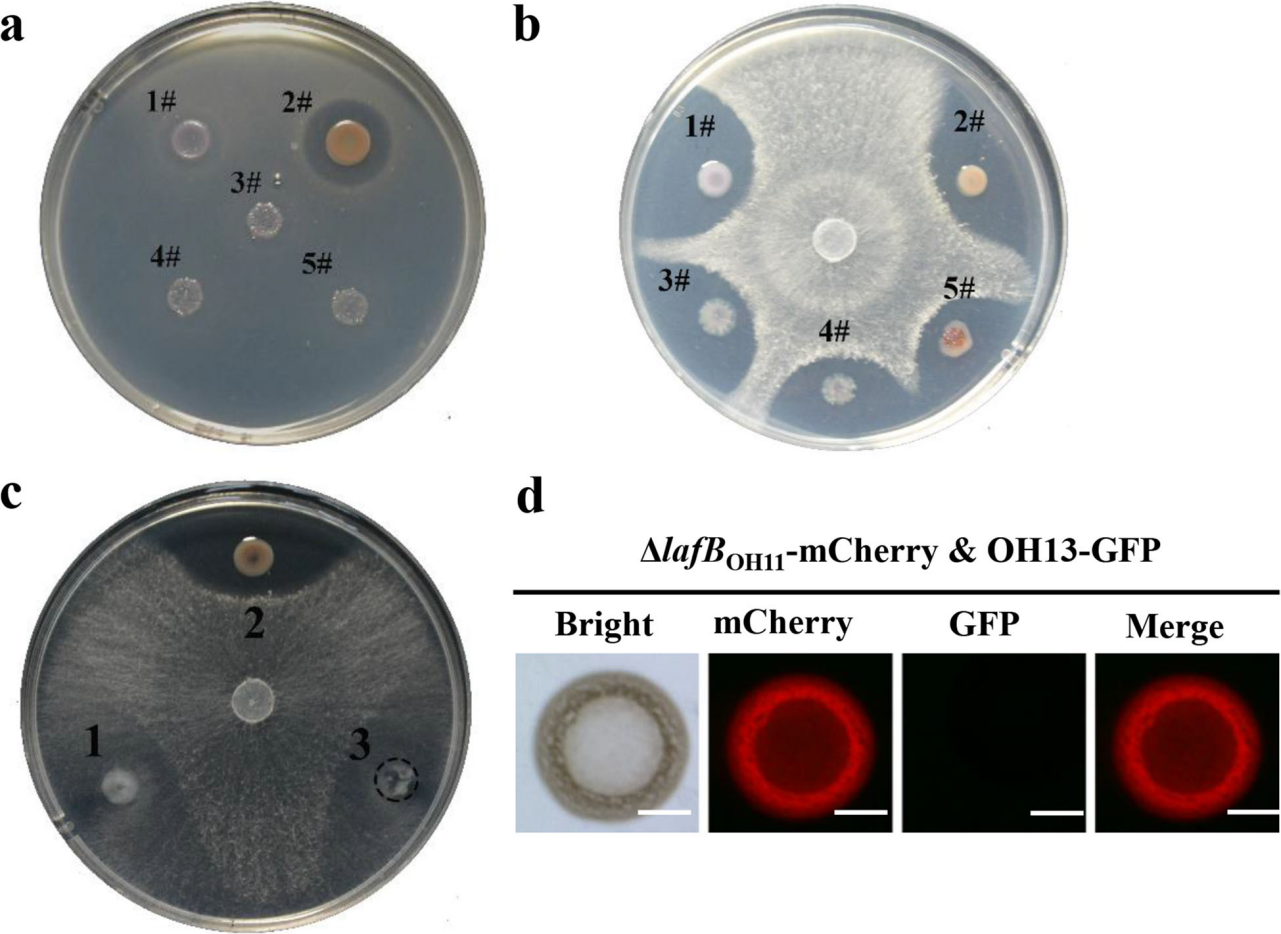
Contact-dependent antibacterial activity locks the natural community of *Lysobacter enzymogenes* OH11 and *Lysobacter antibioticus* OH13 to express synergetic antimicrobial effects. (**a**) Antibacterial test of the OH11-OH13 co-culture on 1/10 TSA plates carrying *E. coli*. The inhibition zone indicates that the inoculated strain has antibacterial activity. 1#, OH11, *L. enzymogenes*; 2, OH13, *L. antibioticus*; 3#-5#, co-culture of OH11 and OH13 at ratios of 1:1 (3#), 1:3 (4#) and 1:5 (5#). (**b**) Antifungal assays of the OH11-OH13 co-culture on 1/10 TSA plates carrying the fungus *Valsa pyri*. The strains numbered 1#-5# are the same as those listed in panel a. (**c**) Antifungal test of co-cultivation of OH13 and OH11-derived mutant strain (Δ*lafB*_OH11_) failing to produce antifungal HSAF. 1#, Δ*lafB*_OH11_, HSAF-deficient mutant of OH11; 2#, OH13; 3#, co-culture of OH13 with Δ*lafB*_OH11_. (**d**) mCherry-labelled Δ*lafB*_OH11_ kills GFP-labelled OH13 through cell-cell contact. The fluorescence signals were observed after 24 h of incubation. The bar represents 2 mm

We embedded the indicator *E. coli* DH5α strain (DH5α) in a 1/10 TSA plates, and inoculated wild-type OH11, OH13 or a mixture of different proportions on the surface of the 1/10 TSA plates carrying DH5α. As expected, the antifungal OH11 failed to inhibit the growth of DH5α, while OH13 can do this by secreting antibacterial factors (Fig. 3a), which is consistent with the previous report (Shen et al., 2021). When OH11 and OH13 were mixed at a ratio of 1:1, 1:3 or 1:5, their co-inoculation could not inhibit the growth of DH5α (Fig. 3a), supporting our hypothesis that the incompatibility between OH13 and OH11 cells restricts their joint use to exhibit the expanded contact-independent antibacterial effect.

We also tested whether the antifungal effect of OH11 would be affected by co-culturing with OH13 on agar plates. As expected, OH11 displayed antifungal activity against *Valsa pyri*, a fungal pathogen causing pear valsa canker (Fig.3b). Although OH13 is mainly used as a contact-independent antibacterial agent, we also found that this strain inhibited the fungal growth (Fig. 3b), which is consistent with the finding described in a previous study (Laborda et al., 2019). Considering OH11 fails to inhibit the growth of gram-negative bacteria, but displays broad-spectrum antifungal activities, due to its production and secretion of an antifungal antibiotic, known as HSAF (Qian et al. 2013). Thus, introducing OH11 to engineer a compatible combination with OH13 is aimed to expand their antimicrobial spectrum or enhance their antifungal activity. However, co-cultivation of OH11 and OH13 at a ratio of 1:1, 1:3 or 1:5 does not seem to visually affect the antifungal activity expressed by each partner (Fig. 3b). It is possible that when OH11 kills OH13 via cell-cell contact in a mixed community, OH11, the dominant bacteria in the community, may be capable of fighting against the selected fungus by secreting a well-characterized antifungal antibiotic called the heat-stable antifungal factor, HSAF (Yu et al. 2007; Qian et al. 2013). In support, the single culture of HSAF-deficient mutant (Δ*lafB*_OH11_) on 1/10 TSA plates failed to inhibit fungal growth, and this phenomenon was also observed when Δ*lafB*_OH11_ was co-inoculated with wild-type OH13 at a ratio of 1:1 on the same plates (Fig. 3c). After 24 h of co-cultivation, the main presence of Δ*lafB*_OH11_ was detected in a mixed community with wild-type OH13 as determined by the florescent microscope (Fig. 3d). In summary, all the above results indicate contact-dependent antibacterial activity blocks *L. enzymogenes* and *L. antibioticus* to show a synergetic antimicrobial effect.

### *L. antibioticus* OH13 carries active T4SS

**Fig. 4:**
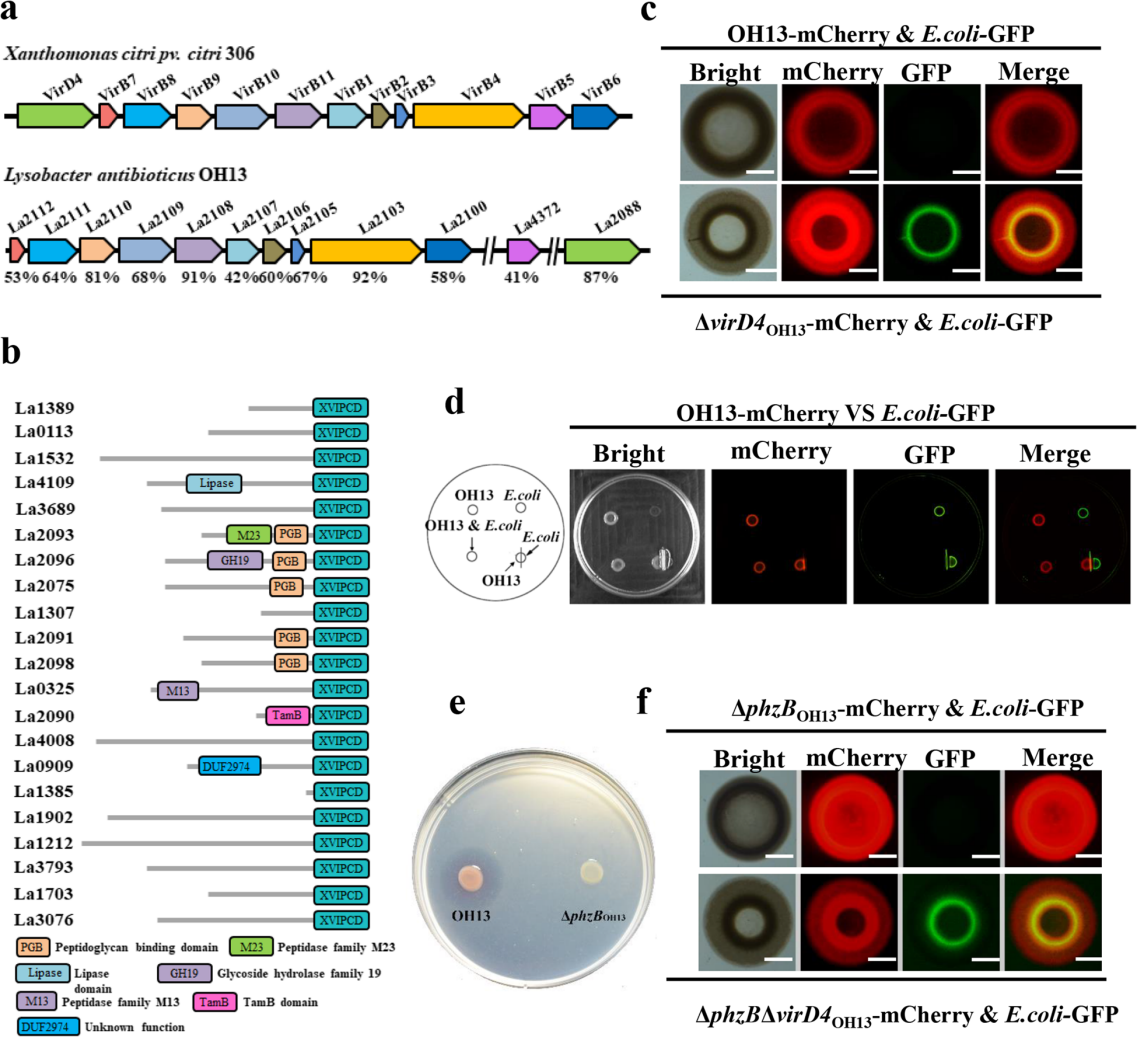
*Lysobacter antibioticus* OH13 carries active T4SS. (**a**) Identification of a complete T4SS gene cluster in *L. antibioticus* OH13. The similarity of each T4SS protein component in OH13 was compared with the respective component of the bacteriacidal T4SS system in *Xanthomonas citri* 306 (Souza et al., 2015). (**b**) Predicted twenty-one T4SS candidate effectors containing the conserved C-terminal XVIPCD domain in the OH13 genome. (**c**) Contact-dependent killing of *E. coli* DH5α (DH5α) by T4SS of OH13. The assay was carried out using mCherry-labelled OH13 or its derivative as the killer strain, while GFP-labelled DH5α was used as the prey. The strains were mixed at a ratio of 1:1 and co-inoculated on 1/10 TSA agar. The fluorescence signals were observed after 24 h of incubation. The bar represents 2 mm. Δ*virD4*_OH13_, an inactivated T4SS deletion mutant of OH13, in which the *virD4* gene encoding T4SS-specific ATPase is deleted in-frame. (**d**) Cell-cell contact is essential for OH13 to kill *E. coli* DH5α. The cell-cell contact of GFP-labelled DH5α and mCherry-labelled OH13 was separated by using a 0.22-μM filter membrane. (**e**) OH13 inhibits the *E. coli* growth by secreting antibacterial phenazine. OH13, wild type; Δ*phzB*_OH13_, an OH13 mutant with disrupted *phzB* gene in the antibacterial phnazine biosynthesis operon. (**f**) Contact-dependent killing of *E. coli* by OH13 using T4SS independent of the antibacterial phenazine production. Δ*phzB*_OH13_, a phenazine-defective mutant of OH13; Δ*phzB*Δ*virD4*_OH13_, Δ*phzB*_OH13_ with an inactivated T4SS due to the in-frame deletion of *virD4* in this strain. The fluorescence signals were observed after 24 h of incubation. The bar represents 2 mm

The above results imply that blocking contact-dependent antibacterial activity seems to be a feasible approach to engineer the OH11-OH13 combination from natural incompatibility to artificial compatibility. A key step for achieving this goal is to understand how contact-dependent antibacterial activity arises between OH11 and OH13. In our earlier studies, we have shown that OH11 possesses both T4SS and T6SS. Among them, T4SS has been experimentally validated as the main contact-dependent bacterial-killing device, but the role of T6SS in bacterial killing remains unknown (Shen et al. 2021; Yang et al. 2020). To test whether the T4SS and/or T6SS is also present in OH13, we first conducted a genomic survey in the genome of OH13. We identified the complete T4SS gene cluster and the existence of 21 predicted T4SS effector proteins, which carry the conserved, C-terminal XVIPCD domain (Fig. 4a-b), but no genes encoding the T6SS structural components could be detected in the genome of OH13. To test whether the predicated T4SS harbored by OH13 is active, we conducted a contact-dependent assay by co-culturing mCherry-labelled OH13 with GFP-labelled *E. coli* DH5α that is model bacteria lacking both T6SS and T4SS (Shen et al. 2021). Results of fluorescent microscopy revealed that when both strains were co-inoculated on a 1/10 TSA plate at a ratio of 1:1, OH13 efficiently killed DH5α (Fig. 4c). We then mutated the *virD4* gene encoding the T4SS-specific ATPase in OH13 through the in-frame deletion approach and found that the mCherry-labelled mutant (Δ*virD4*_OH13_) failed to kill the GFP-labelled DH5α (Fig. 4c), suggesting that OH13 carrying an active T4SS mediates bacterial killing via cell-cell contact. To support this conclusion, we first separated the growth of mCherry-labelled OH13 and GFP-labelled DH5α by a 0.22-μM filter membrane. This step resulted in the failure of OH13 to kill DH5α (Fig. 4d), revealing that the cell-cell contact is required for the function of the T4SS device possessed by OH13. To support the specific role of T4SS in the observed contact-dependent killing of DH5α by OH13, we selected the available antibacterial phenazine-defective mutant Δ*phzB*_OH13_ (Zhao et al. 2016). On the 1/10 TSA plates, this mutant (Δ*phzB*_OH13_) cannot inhibit the growth of DH5α because it lacks the secretion of antibacterial phenazines antibiotics (Fig. 4e). However, the fluorescent microscope clearly showed that when the two strains were mixed at a ratio of 1:1 and co-inoculated on 1/10 TSA plate, mCherry-labelled Δ*phzB*_OH13_ also effectively killed GFP-labelled DH5α (Fig. 4f). Moreover, when GFP-labelled DH5α and mCherry-labelled Δ*phzB*_OH13_ with *virD4* mutation were co-cultivated at the same ratio, the contact-dependent killing effect disappeared (Fig. 4f). These results collectively suggest that OH13 carries active T4SS and mediates contact-dependent bacterial killing.

### The contact-dependent antibacterial activity occurring in the interaction between *L. enzymogenes* and *L. antibioticus* is mainly determined by T4SS

The discovery of active T4SS in both OH11 and OH13 promoted us to explore whether T4SS mediates their naturally-occurring contact-dependent antibacterial activity observed in Fig. 1. For this purpose, mCherry-labelled OH11 and GFP-labelled OH13 were mixed at various ratios of 1:1, 1:3, 1:5, 1:10, 1:20 and 1:50. After co-inoculation on 1/10 TSA plates for 24 h, we were surprised to find that in all the tested co-culture samples, mCherry-labelled OH11 always effectively killed GFP-labelled OH13 (Fig. S1). But when their growth was separated by a 0.22-μM filter membrane, no killing of OH13 by OH11 was observed (Fig. 2), suggesting that cell-cell contact is essential for the observed contact-dependent antibacterial activity between OH11 and OH13.

**Fig. 5:**
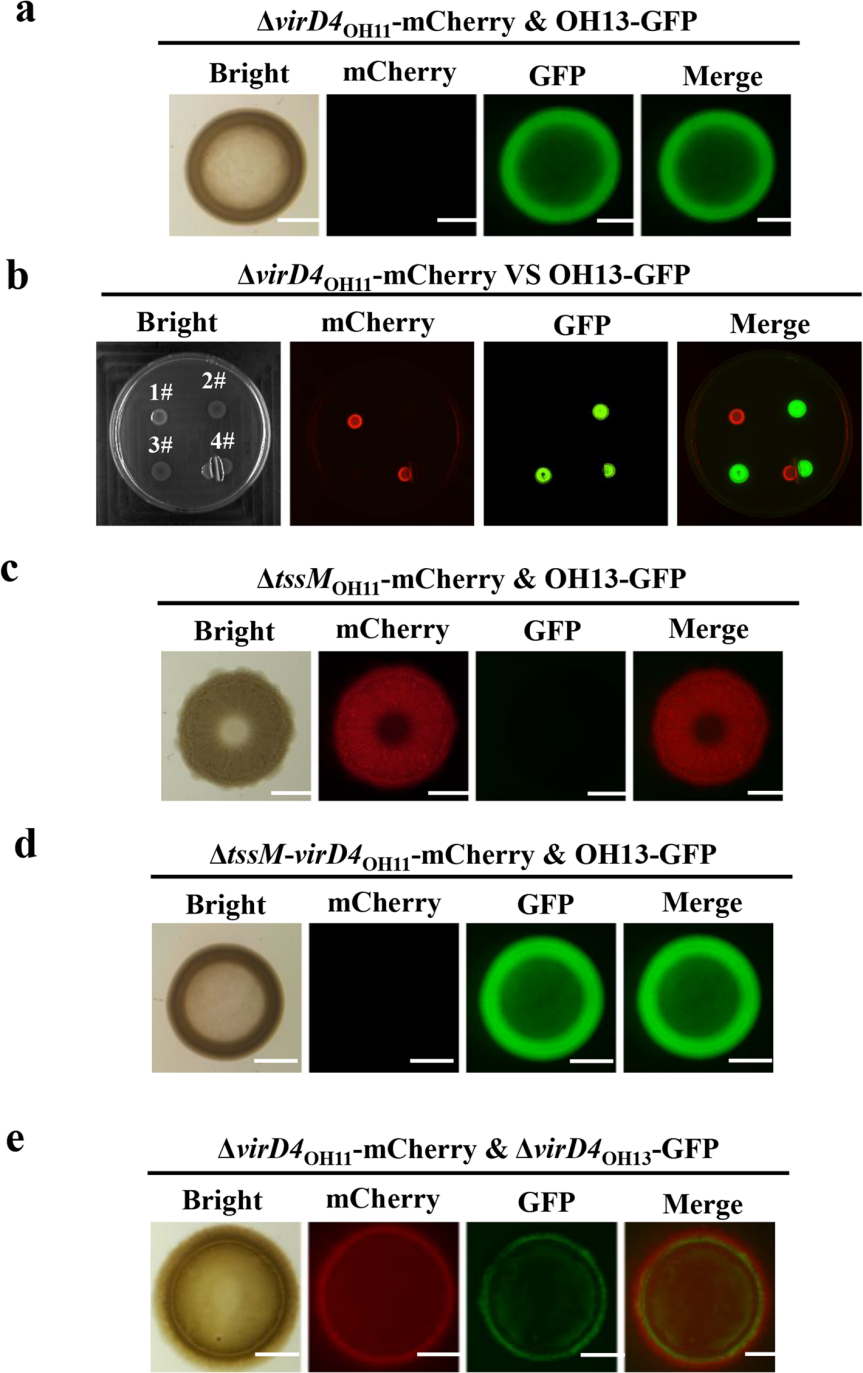
Inactivating the T4SS of both *Lysobacter enzymogenes* OH11 and *Lysobacter antibioticus* OH13 achieves the artificial compatibility of each other. (**a**) GFP-labelled OH13 kill mCherry-labelled OH11 with inactivated T4SS. Δ*virD4*_OH11_, an ascertained T4SS-inactivated mutant of OH11 (Shen et al., 2021). The fluorescence signals were observed after 24 h of incubation. The bar represents 2 mm. (**b**) Cell-cell contact is essential for killing mCherry-labelled Δ*virD4*_OH11_ by GFP-labelled OH13. A 0.22-μM filter membrane was used to separate cell-cell contact of OH13 and Δ*virD4*_OH11._ 1#,Δ*virD4*_OH11_; 2, OH13; 3#, Δ*virD4*_OH11_-OH13 (1:1 ratio); 4#, separation of Δ*virD4*_OH11_ and OH13 by filter membrane. (**c**) Contact-dependent killing of OH13 by OH11 with inactivated T6SS. Δ*tssM*_OH11_, an ascertained T6SS-inactivated mutant (Yang et al., 2020). (**d**) T4SS, but not T6SS, is the key for the contact-dependent killing of OH13 by OH11. Δ*tssM*_OH11_, an ascertained T6SS-inactive mutant; Δ*tssM*Δ*virD4*_OH11_, Δ*tssM*_OH11_ with inactivated T4SS. (**e**) By double inactivating T4SS in the two strains, cell-cell compatibility between OH11 and OH13 can be engineered, T4SS-inactivated mutants of OH11 and OH13, designated as Δ*virD4*_OH11_ and Δ*virD4*_OH13,_ respectively, were co-cultivated on 1/10 TSA agar at a ratio of 1:1. The co-existence of both engineered strains in the mixed colony is indicated by observing the fluorescent signals from mCherry and GFP. In panel c-e, fluorescence signals were observed after 24 h of incubation. The bar represents 2 mm

Is T4SS-active OH13 attacked by OH11 using the same apparatus? To test this, the T4SS inactivated mutant Δ*virD4*_OH11_ and wild-type OH13 was mixed at a ratio of 1:1 and co-cultivated on 1/10 TSA plate. We observed that GFP-labelled OH13 almost completely killed mCherry-labelled Δ*virD4*_OH11_ (Fig. 5a). This phenomenon depends on their cell-cell contact, because when the growth of OH13 and Δ*virD4*_OH11_ was separated by filter membrane, the observed killing disappeared again (Fig. 5b). These findings indicate that if there is no T4SS device, OH11 seems to be counter-attacked by OH13 using the T4SS. To confirm this conclusion, we provided two additional pieces of evidence. We first show that under similar co-culture conditions, mCherry-labelled, OH11-derivative strain (Δ*tssM*_OH11_) with inactivated T6SS, such as wild-type OH11, still effectively kill GFP-labelled OH13 (Fig. 5c). In the context of Δ*tssM*_OH11_ background, the in-frame deletion of *virD4* caused the mCherry-labelled double mutant (Δ*tssM*-*virD4*_OH11_) to be almost killed by GFP-labelled OH13 (Fig. 5d), supporting the conclusion that T4SS in OH11 served as an attack-defense device during its cell-cell interaction with T4SS-producing OH13.

Next, we tested what happens when the cells of the OH11 T4SS mutant are in contact with the cells of the OH13 T4SS mutant. We found that mCherry-labelled Δ*virD4*_OH11_ and GFP-labelled Δ*virD4*_OH13_ established a compatible cell-cell interaction. When they were co-cultivated on 1/10 TSA plate at a ratio of 1:1, the two mutant strains were found to co-exist (Fig. 5e). Together, above results suggest that the T4SS device mediates the process of attack and counterattack between OH11 and OH13 cell-cell interactions, and that double blocking of this system in the two strains enable them to switch their cellular interactions from natural incompatibility to artificial compatibility.

### Blocking contact-dependent antibacterial activity by double-inactivating T4SS unlocks the synergistic contact-independent antimicrobial effect co-expressed by *L. enzymogenes* and *L. antibioticus*

**Fig. 6:**
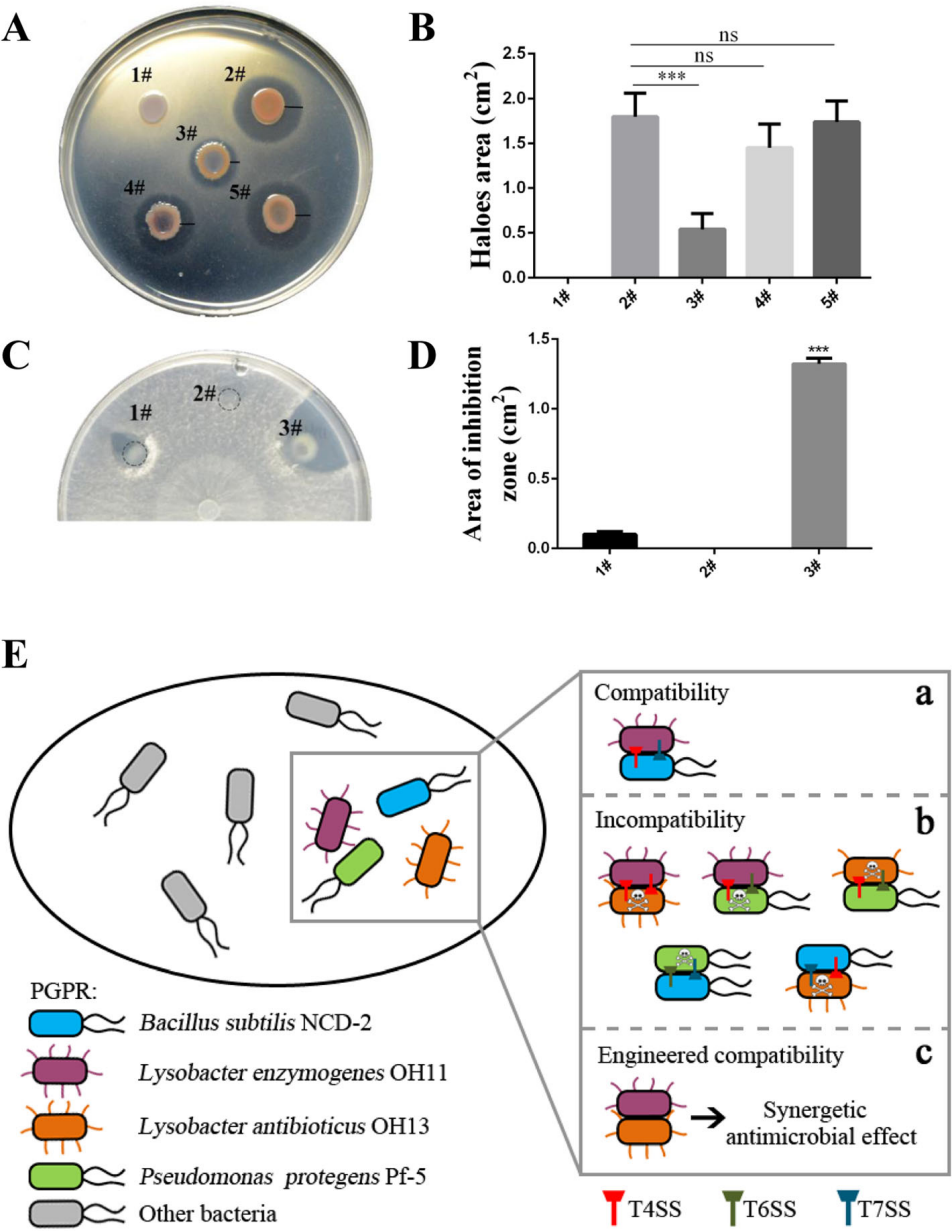
The artificial compatible *Lysobacter enzymogenes* OH11 and *Lysobacter antibioticus* OH13 shows synergetic antimicrobial effects. (**a**) Inhibition test of single culture of Δ*virD4*_OH11_, Δ*virD4*_OH13_ or their combination against *E. coli* DH5α (DH5α). The inhibition zone indicates that the inoculated strains have antibacterial activity against DH5α. 1#, Δ*virD4*_OH11_, the T4SS-inactivated mutant of *L. enzymogenes* OH11; 2, Δ*virD4*_OH13_, the T4SS-inactivated mutant of *L. antibioticus* OH13; 3#-5#, co-culture of Δ*virD4*_OH11_ and Δ*virD4*_OH13_ at ratios of 1:1 (3#), 1:3 (4#) and 1:5 (5#). Bars represent the radius of the zones. (**b**) The statistical analysis of the antibacterial zones in panel a. The antibacterial zone was determined by the formula of π × R^2^. The mean ± standard deviation of three replicates for each treatment is represented by the column. Asterisks indicate values that are significantly different according to the Student’s t-test (α = 0.01). (**c**) Antifungal test of Δ*virD4*_OH11_-Δ*virD4*_OH13_ co-culture on agar plates carrying the fungus *Valsa pyri*. The Δ*virD4*_OH11_ or Δ*virD4*_OH13_ strain was grown in LB to an OD_600_ of 1.0, and then diluted 1000 folds. The obtained diluted culture of each strain was inoculated on the surface of agar plates alone or in combination at a ratio of 1:1. 1#, Δ*virD4*_OH11_; 2, Δ*virD4*_OH13_; 3#, co-culture of Δ*virD4*_OH11_ and Δ*virD4*_OH13_ at a ratio of 1:1. (**d**) The statistical analysis of the antifungal zones in panel c. The inhibition zones were determined and averaged as the radius R according to an earlier report (Yang et al. 2020) using the formula π × R^2^. The mean ± standard deviation of three replicates for each treatment is represented by the column. ***P < 0.001 relative to 1#. (**e**) A biotechnology strategy that demonstrates how to unlock contact-dependent inhibition in the cell-cell interactions of selected soil-borne biocontrol bacteria. In the soil microbiome, various biocontrol bacteria co-inhabit, and rely on contact interactions to occur continuously. Of these interactions, some are compatible, as demonstrated by the OH11-NCD-2 pair (**A**), while some interactions are incompatible, causing one species to kill the other through cell-cell contact (represented by the skeleton symbol) (**B**). The occurrence of these natural contact-dependent inhibitions may be due to the existence of multiple contact-dependent killing devices, such as T4SS in OH11 and OH13, T6SS in Pf-5, and T7SS assumed in NCD-2 (**B**). Modifying these contact-dependent killing devices by deleting some T4SS genes (i.e. *virD4*), such as the T4SS in both OH11 and OH13, proved to be effective in transforming the two-species interaction from natural incompatibility to artificial compatibility. Thereby, it is reasonable to generate a synergetic and improved antimicrobial effect of engineered biocontrol alliance (**C**)

Since the double blockade of T4SS in OH11 and OH13 unlocked their contact-dependent antibacterial activity, we tested whether this genetic engineering is suitable for designing artificial and synergetic biocontrol community. To this end, we again embedded the indicator *E. coli* DH5α in the 1/10 TSA plates, and inoculated Δ*virD4*_OH11_, Δ*virD4*_OH13_ or their mixture with various ratios (1:1, 1:3 and 1:5) on the surface of the 1/10 TSA plate carrying DH5α. We found that, like the wild-type OH11, the Δ*virD4*_OH11_ strain failed to exhibit an inhibitory zone against DH5α, while the OH13-derivative and T4SS-inactivated mutant Δ*virD4*_OH13_ did (Fig. 6a), suggesting that inactivation of the T4SS in OH13 does not impair its natural antibacterial function. Unlike the OH11-OH13 wild-type pair, all co-cultures of Δ*virD4*_OH11_ and Δ*virD4*_OH13_ with the above three selected ratios displayed an inhibitory zone against DH5α (Fig. 6a-b), revealing that double blockade of T4SS in OH11 and OH13 indeed enables their co-existence to exhibit contact-independent antibacterial ability together. Further, through the 1000-fold dilution of the bacterial cultures, we further observed that the co-culture of T4SS mutant strains (Δ*virD4*_OH11_ and Δ*virD4*_OH13_) on 1/10 TSA plate displayed significantly enhanced antifungal effect compared to their single cultures (Fig. 6c-d). These finding collectively suggest that it is feasible to unlock the contact-dependent antibacterial activity between OH11 and OH13 by co-inactivating T4SS to engineer artificial biocontrol community with expanded biocontrol spectrum and enhanced antimicrobial activity.

## Discussion

Although more and more evidences show that many animal and plant pathogenic bacteria use contact-dependent antibacterial activity to compete with each other to gain advantages in ecological adaption, survival and host infection (Trunk et al. 2018), the interactions between cells and plant-beneficial bacteria has not been well resolved. In the present study, we show that the bacterial contact-dependent antibacterial activity is common among interactions of several selected PGPR representatives that are known as the major members of biocontrol bacteria (Fig. 1; Mendes et al., 2013). These naturally-occurring contact-dependent antibacterial activities are likely to restrict the two-species community that express synergetic biocontrol effects, as evidenced by the observation that contact-dependent antibacterial activity between the antifungal *L. enzymogenes* and the antibacterial *L. antibioticus* locks their wild-type strains to jointly exhibit an antibacterial effect (Fig. 3a). The contact-dependent antibacterial activity between these two biocontrol *Lysobacter* species is jointly determined by the newly-discovered, bacteria killing device (called T4SS). Both *L. enzymogenes* and *L. antibioticus* use active T4SS to attack and counter-attack each other. By jointly inactivating T4SSs in the two species to prevent T4SS-mediated bacterial warfare in the cell-to-cell interaction of biocontrol agents, it is possible to unlock their natural incompatibility, thereby rationally engineering an artificial two-species biocontrol alliance to co-express antifungal and contact-independent antibacterial activity (Fig. 6e). Therefore, this study highlights a feasible and simple approach that can promote microbiologists to design synergistic biocontrol communities by modifying bacterial contact-dependent antibacterial activity. This approach is also suitable for designing a range of biocontrol communities containing multiple species, because contact-dependent killing devices such as T4SS, T6SS and T7SS are widely distributed in biocontrol agents (Souza et al., 2015; Trunk et al., 2018; Bottai et al., 2016).

The design of bacterial contact-dependent antibacterial activity by inactivating the contact-dependent killing devices also provides a potentially novel approach for plant microbiome engineering. The plant microbiome provides fitness advantages to plants by promoting growth, facilitating nutrient uptake, improving stress tolerance, and enhancing resistance to pathogens (Mendes et al., 2013). These diverse and beneficial effects expressed by the plant microbiome prompt scientists to artificially select plant microbial communities to design evolutionary microbiome functions that promote plant health and fitness (Mueller and Sachs 2015). A widely documented approach applied to plant microbiome engineering is to use plant phenotypes as probes to measure and manipulate those microbiomes that have specific and beneficial effects on plant health (Mueller and Sachs 2015). Since PGPR is a core member of plant microbiome, we believe that bacterial contact-dependent antibacterial activity highlighted in this study can be used as an additional universal probe to rationally design plant microbiomes.

T4SS is designed to achieve the co-existence of *L. enzymogenes* and *L. antibioticus* for biocontrol from cell-to-cell incompatibility to compatibility. This is the first case of transforming the basic research of bacterial contact-dependent antibacterial activity into applied microbiology. This translation process may also be applicable to unlock the incompatible cell-to-cell interactions between the T4SS- and T6SS-active bacteria. A recent work in our laboratory proved that *L. enzymogenes* OH11 producing T4SS can effectively kill the antibacterial *Pseudomonas protegens* Pf-5 with T6SS activity, causing their wild-type community to be incompatible. When T4SS in *L. enzymogenes* and T6SS in *P. protegens* Pf-5 are doubly inactivated, their mutant community becomes compatible (Shen et al. 2021).

The observed compatible and incompatible cell-cell interactions between PGPR species also provide valuable clues to uncover the underlying mechanisms for these PGPRs to establish interesting cell-cell competition or recognition patterns in the future. First, the observed killing of *L. antibioticus* OH13 by *L. enzymogenes* OH11 via cell-cell contact raises an attractive fundamental question, namely, why T4SS active OH11 gains a competition advantage over OH13 that also carries an active T4SS. Although wild-type OH11 uses T4SS to kill OH13, the results from the T4SS inactivation assay clearly show that wild-type OH13 is also visually effective in killing the OH11 T4SS-inactivated mutant Δ*virD4*_OH11_. This finding suggests that both wild-type OH11 and OH13 use functional T4SS to manage their cell-cell interactions in an attack-counterattack manner. Although uncovering the underlying mechanisms is not the focus of this study, similar findings have been previously reported between the cell-cell interactions of two T6SS-postive bacteria, *Pseudomonas aeruginosa* and *Vibrio cholerae*, in which the former targets the latter for T6SS-mediated counterattack (Basler et al. 2013). Further mechanistic investigations have shown that *P. aeruginosa* “smartly” adopts a unique ‘Tit-for-Tat’ evolutionary strategy to control its cell-cell interactions with *V. cholerae*. By using this strategy, the *P. aeruginosa* T6SS organelle assembly and lethal counterattack via delivery of a toxic Tse1 effector into the periplasm of *V. cholerae* are regulated by a signal that corresponds to the point of attack of *V. cholerae* using its functional T6SS (Basler et al. 2013). Whether the intercellular interaction between T4SS-positve OH11 and OH13 also involves the ‘Tit-for-Tat’ strategy remains unknown and is deserved for future investigation. We also could not exclude the possibility that the contact-dependent interspecies killing of the T4SS-active OH13 by OH11 is due to the presence of one or more unique toxic T4SS effector genes encoded by the genome of OH11. Second, it is surprising to observe the contact-dependent co-existence of *L. enzymogenes* OH11 (but not OH13) and *Bacillus subtilis* NCD-2, because a previous study showed that T4SS-positive *Xanthomonas citri* can inject a toxic effector protein X-Tfe^XAC2609^ to lyse a major component of the *Bacillus* cell wall, called peptidoglycan (Souza et al. 2015). Therefore, the natural compatibility of OH11-NCD-2 reveals the first naturally occurring, compatible interaction between gram-negative, T4SS-active (*Lysbacter* sp.) and gram-positive, T7SS-active (*Bacillus* sp.) bacterium. Interestingly, such a compatible, interspecies interaction seems to be common, because we found that *L. enzymogenes* OH11 also established compatible cell-cell interactions with *B. subtilis* 168 (168), a model strain of *Bacillus* (Fig. S2). These findings suggest that *L. enzymogenes* may possess an uncharacterized cell-cell recognition mechanism to control its compatible interactions with *Bacillus* spp.

## Conclusions

Here, we demonstrated for the first time that by co-inactivating T4SS to unlock bacterial contact-dependent antibacterial activity, it is particle to engineer a two-species alliance with synergetic biocontrol effects. This contact-dependent antibacterial activity-mediated engineering clarifies the rational design of bacterial biocontrol communities in agriculture. We show that one cannot just randomly combine single high-performance biocontrol agents together to generate a synergistic two-species community. Knowing in advance on their contact-dependent compatibility seems to be a key step. This understanding is also valuable for the rational joint use of commercial bio-pesticides based on living cell to avoid their potential intercellular killing events.

## Methods

### Bacterial strains, plasmids and growth conditions

The bacterial strains and plasmids used in this work are listed in supplemental Table S1. Unless otherwise specified, *L. enzymogenes* strain OH11 (CGMCC No. 1978), *L. antibioticus* OH13 (CGMCC No.7561) and their derivatives were grown in Luria-Bertani (LB) at 28 °C. Kanamycin (Km, 25 μg/mL) was added to the media to generate mutants, and gentamicin (Gm, 150 μg/mL) was used to maintain the plasmid. The *Escherichia coli* strains and *Bacillus subtilis* strains (NCD-2 and 168) were grown in LB medium at 37 °C, while *Ps. protegens* Pf-5 were grown in the same medium at 28 °C.

### Genetic methods

As mentioned earlier, approaches involving double-crossover homologous recombination and marker exchange were used to generate in-frame destruction mutants in OH13 (Zhao et al. 2016). In brief, ~ 1000-bp fragments homologous to the upstream and downstream regions of the target gene were amplified by PCR with specific primers (Table S2). The kanamycin cassette amplified from the vector pET30 (Table S1) was ligated with these two fragments and cloned into the suicide vector pJQ200SK (Zhao et al. 2016). This resulting vector was transferred into *E. coli* S17–1 and further to the OH13 recipients by conjugation. To select candidate gene-deletion mutants, the transconjugants were plated on LB plate containing 10% (w/v) sucrose, 100 μg/mL Amp, and 50 μg/mL Km. Positive mutants were verified by PCR using specific primers (Table S2).

### Bioinformatics analyses

The T4SS structural proteins from the phylogenetic-related bacterial strain *X. citri* 306 (NC_003919.1) were used as a query to run local BLASTp to identify the corresponding homologs in the OH13 genome. When the E-value is lower than 10^− 5^ and the similarity percentage with the corresponding *X. citri* 306 homologous protein is higher than 35%, the protein is considered to be present. To predict the presence of XVIPCD-domain proteins in OH13, the XVIPCD domain sequences of 13 *X. citri* XVIPCD proteins from *X. citri* 306 (Souza et al. 2015) were first aligned by the MUSCLE tool, and then used to construct a profile of Hidden Markov Model (HMM), followed by the HMM search against OH13 proteins using the hmmsearch program implemented in HMMER (Finn et al. 2011). A XVIPCD domain was considered to be present when the HMM search E-value is lower than 10^− 5^.

### Contact-dependent killing assay

The fluorescence-mediated, contact-dependent killing assays were performed according to a procedure previously described in the laboratory with some modifications (Shen et al. 2021). In brief, the plasmid pYC12 carrying the mCherry gene driven by the plasmid constitutive promoter (P_*tac*_) was introduced into the *Lysobacter* strains. The plasmid pBBR1 containing the constitutively expressed GFP gene was transferred to *E. coli* DH5α, OH13, and its mutants. The GFP-labelled strains of *B. subtilis* NCD-2 and 168 were produced by early works (Dong et al. 2020) and were kindly donated by Prof. Ping Ma (Hebei Academy of Agricultural and Forestry Sciences, China) and Prof. Huijun Wu (Nanjing Agricultural University, China), respectively. GFP-labelled *Pseudomonas protegens* Pf-5 was previously generated and storied in the laboratory (Shen et al. 2021). After incubating overnight in LB medium at 28 °C in an orbital shaker (200 rpm), all bacterial cells were collected by centrifugation (6000 rpm for 3 min at room temperature) and suspended in fresh LB to reach the final OD_600_ of 1.0. A volume of 750 μL of the resultant cell suspension of the following bacterial combinations was mixed either equally or by other ratios as mentioned in the manuscript: OH11-OH13; NCD-2-Pf-5; OH13-NCD-2; OH13-Pf-5, and OH11-NCD-2. After that, 5 μL of the mixed culture was spot-inoculated on 1/10 TSA dishes, followed by incubation at 28 °C for 24 h. A 0.22-μM filter membrane was inserted into the 1/10 TSA agar plate. Then 5 μl of the two bacterial cultures were respectively spotted on the plate beside the membrane. A stereoscopic fluorescence microscope (Nikon SMZ25, Nikon, Japan) was used to observe the fluorescence signal. GFP and mCherry fluorescence were excited at 488 nm and 561 nm, respectively. All experiments were carried out three times with three replicates for each treatment.

### Antifungal and antibacterial assays

In the fungal inhibition assay, a plug (2 mm diameter) cut from the border of a 5-day old colony of the soilborne fungal pathogen *V. pyri* SXYL134 (Table S1) was transferred from Potato Dextrose Agar to the centre of dishes of 1/10 Trytpic Soy Broth (TSB) agar. Subsequently, 2 μL of OH11 and OH13 cell suspension (OD_600_, 1.0), alone or in combination (1:1, 1:3, 1:5 mixture), was inoculated on the edge of dishes previously inoculated with *V. piri*. The antagonistic activity was indicated by the inhibition zones around the colonies after 3 days of incubation at 28 °C. The area of the antifungal zone was calculated using the following formula: area = π × (radius)^2^, where the radius is the average value of the longest axis and the shortest axis of the inhibition zones as described previously (Yang et al., 2020; Shen et al. 2021). All experiments were carried out three times with three replicates for each treatment. In the bacterial inhibition assay, 1 mL of the overnight culture of indicator strain *E. coli* DH5α was mixed with 100-mL 1/10 TSA medium and poured into a Petri dish. Once solidified, 5 μL of the OH11 and OH13 cell suspension (OD_600_, 1.0) was spot-inoculated alone or in combination (1:1, 1:3, 1:5 mixture) onto the surface of 1/10 TSA culture dishes containing *E. coli* DH5α. After 3 days of incubation at 28 °C, a Nikon camera (D7100, Japan) was used to photograph the inhibition zones in the antibacterial assay. The area of the antibacterial zone was calculated using the following formula: area = π × (radius)^2^, where the radius is the average value of the inhibition zones as described previously (Shen et al. 2021). All experiments were carried out three times with three replicates for each treatment. The mean values were compared using the Student’s T test (α = 0.05) implemented in the SPSS 14.0 software package (SPSS Inc., Chicago, IL, USA).

## Supplementary Information

**Additional file 1: Table S1.** Strains and plasmids used in this study. Table S2 Primers used in this study. Fig. S1 Fluorescence evaluation of contact-dependent antibacterial activity event by co-cultivating *L. enzymogenes* OH11 and *L. antibioticus* OH13 on 1/10 TSA agar at various ratios. The fluorescence signals were observed after 24 h of incubation. Wild-type OH11 and OH13 were labelled by mCherry and GFP, respectively. The selected co-cultivation ratios are shown. Fig. S2 Compatible cell-cell interaction between *L. enzymogenes* OH11 and *Bacillus subtilis* 168. Wild-type OH11 and 168 were labelled by mCherry and GFP, respectively. The cultures of both strains were mixed at a ratio of 1:1 and co-incubated on 1/10 TSA agar. The fluorescence signals were observed after 24 h of incubation.

## Data Availability

All strains, plasmids, genes and proteins mentioned in this study are included in this manuscript.
