# Supplementary material for: Unlocking the bacterial contact-dependent antibacterial activity to engineer a biocontrol alliance of two species from natural incompatibility to artificial compatibility
Source: Stress Biol. 2021 Dec 16;1(1):19. doi: 10.1007/s44154-021-00018-x (PMC10441968; doi:10.1007/s44154-021-00018-x)
Supplement: Supplementary file 1 — Additional file 1: Table S1. Strains and plasmids used in this study. Table S2 Primers used in this study. Fig. S1 Fluorescence evaluation of contact-dependent antibacterial activity event by co-cultivating L. enzymogenes OH11 and L. antibioticus OH13 on 1/10 TSA agar at various ratios. The fluorescence signals were observed after 24 h of incubation. Wild-type OH11 and OH13 were labelled by mCherry and GFP, respectively. The selected co-cultivation ratios are shown. Fig. S2 Compatible cell-cell interaction between L. enzymogenes OH11 and Bacillus subtilis 168. Wild-type OH11 and 168 were labelled by mCherry and GFP, respectively. The cultures of both strains were mixed at a ratio of 1:1 and co-incubated on 1/10 TSA agar. The fluorescence signals were observed after 24 h of incubation. [file 44154_2021_18_MOESM1_ESM.docx]

**Supplemental materials**

**Unlocking the bacterial contact-dependent inhibition to engineer a biocontrol alliance of two species from natural incompatibility to artificial compatibility**

Qianhua Wu^a^, Bozhen Wang^a^, Xi Shen^a^, Danyu Shen^a^, Bingxin Wang^a^, Qinggang Guo^b^, Tao Li^c^ , Shan-Ho Chou^d^, Xiaolong Shao^a^, and Guoliang Qian^a*^

^a^College of Plant Protection, Laboratory of Plant Immunity, Key Laboratory of Integrated Management of Crop Diseases and Pests, Nanjing Agricultural University, No.1 Weigang, Nanjing, Jiangsu 210095, PR China

^b^Institute of Plant Protection, Hebei Academy of Agricultural and Forestry Sciences, Integrated Pest Management Center of Hebei Province, Key Laboratory of IPM on Crops in Northern Region of North China, Ministry of Agriculture and Rural Affairs of China, Baoding 071000, PR China

^c^Shanghai Veterinary Research Institute, Chinese Academy of Agricultural Sciences, Shanghai 200241, PR China

^d^Institute of Biochemistry, and NCHU Agricultural Biotechnology Center, National Chung Hsing University, Taichung, Taiwan, ROC

^*^**Correspondence to:** glqian@njau.edu.cn

**Table S1. Strains and plasmids used in this study**

| **Strains and plasmids** | **Characteristics^a^** | **Source** |
| --- | --- | --- |
| *Lysobacter enzymogenes* | | |
| OH11 | Wild type, Km^R^ | Qian et al. 2009 |
| Δ*virD4* _OH11_ | In-frame deletion of *virD4*, Km^R^ | Shen et al. 2021 |
| Δ*tssM* _OH11_ | In-frame deletion of *tssM*, Km^R^ | Yang et al. 2020 |
| Δ*tssM*-*virD4*_OH11_ | In-frame deletion of both *tssM* and *virD4*, Km^R^ | This study |
| Δ*lafB* | In-frame deletion of *lafB*, Km^R^ | Wang et al. 2017 |
| OH11-mCherry | OH11 harboring plasmid pYC12-mCherry, Gm^R^, Km^R^ | Shen et al. 2021 |
| Δ*virD4* _OH11_-mCherry | Δ*virD4*_OH11_ harboring plasmid pYC12-mCherry, Gm^R^, Km^R^ | Shen et al. 2021 |
| Δ*tssM* _OH11_-mCherry | Δ*tssM* _OH11_ harboring plasmid pYC12-mCherry, Gm^R^, Km^R^ | This study |
| Δ*tssM*-*virD4*_OH11_-mCherry | Δ*tssM*-*virD4*_OH11_ harboring plasmid pYC12-mCherry, Gm^R^, Km^R^ | This study |
| Δ*lafB*-mCherry | Δ*lafB*  harboring plasmid pYC12-mCherry, Gm^R^, Km^R^ | Shen et al. 2021 |
| *Lysobacter antibioticus* | | |
| OH13 | Wild type, Amp^R^ | Zhao et al. 2016 |
| Δ*virD4* _OH13_ | Deletion mutant of *virD4*, Km^R^, Amp^R^ | This study |
| Δ*phzB* | In-frame deletion of *phzB*, Amp^R^ | Zhao et al. 2016 |
| Δ*phzB- virD4* | Deleting *virD4* in Δ*phzB*, Km^R^, Amp^R^ | This study |
| OH13-mCherry | OH13 harboring plasmid pYC12-mCherry, Gm^R^, Amp^R^ | This study |
| Δ*virD4* _OH13_-mCherry | Δ*virD4* _OH13_ harboring plasmid pYC12-mCherry, Gm^R^, Km^R^, Amp^R^ | This study |
| Δ*phzB*-mCherry | Δ*phzB*  harboring plasmid pYC12-mCherry, Gm^R^, Amp^R^ | This study |
| Δ*phzB- virD4*-mCherry | Δ*phzB-virD4* harboring plasmid pYC12-mCherry, Gm^R^ , Km^R^, Amp^R^ | This study |
| OH13-GFP | OH13 harboring plasmid pBBR1-GFP, Gm^R^, Amp^R^ | This study |
| Δ*virD4* _OH13_-GFP | Δ*virD4*_OH13_ harboring plasmid pBBR1-GFP, Gm^R^, Km^R^ | This study |
| *Escherichia coli* | | |
| DH5α | Host strain for molecular cloning | Lab collection |
| S17-1 | *Λpir pro hsdR*, *recA* | Lab collection |
| *E.coli*-GFP | *E.coli* DH5α harboring pBBR1-GFP vector, Gm^R^ | This study |
| Other bacteria | | |
| NCD-2-GFP | *Bacillus subtilis* NCD-2 harboring pC-1-GFP vector, Chl^R^ | Dong et al. 2020 |
| Pf-5-GFP | *Pseudomonas protegens* Pf-5, GFP-labelled strain, Amp^r^, Km^r^ | Shen et al. 2021 |
| Pf-5-mCherry | *Pseudomonas protegens* Pf-5, mCherry-labelled strain, Amp^R^, Gm^R^ | A gift strain from Prof. Huijun Wu |
| Fungus | | |
| *Valsa pyri* SXYL134 | A fungal pathogen causing pear Valsa canker | Yin et al., 2015 |
| Plasmids | | |
| pJQ200SK | Suicide vector with a *sacB* gene, Gm^R^ | Quandt and Hynes 1993 |
| pJQ200SK-*virD4*_OH13_-KM | pJQ200SK with two flanking fragments of *virD4*_OH13_ ligated with a kanamycin cassette, Gm^R^, Km^R^ | This study |
| pET30 | Protein expression vector with a His tag, Km^R^ | Wang et al. 2018 |
| pBBR1-GFP | pBBR1-MCS5 containing the coding region of GFP, Gm^R^ | Shen et al. 2021 |
| pYC12-mCherry | pYC12 containing the coding region of mCherry, Gm^R^ | Ling et al. 2016 |

^a^Km^R^, Gm^R^ Amp^R^, kanamycin, gentamicin, ampicillin, resistance, respectively.

**Table S2. Primers used in this study**

| **Primer** | **Sequence (5'-3')^a^** | **Purpose** |
| --- | --- | --- |
| *virD4*_OH13_-F1 | TTCCTGCAGCCCGGGGGATCCCGGGCGCCTCGCCCTTGT | To amplify a 1052-bp fragment upstream of *virD4*_OH13_ |
| *virD4*_OH13_-R1 | GCTATACGAACGGTACATCAACGTCACGTATCCCG |  |
| *virD4*_OH13_-F2 | ATTATACGAACGGTACCGGTGAAATGCGACAAGAT | To amplify a 1053-bp fragment downstream of *virD4*_OH13_ |
| *virD4*_OH13_-R2 | AGCTCCACCGCGGTGGCGGCCGCCTTGCGCCGCTCCTACCC |  |
| KM-F | TACCGTTCGTATAGCATACATTATACGAAGTTATGAAGCTCCCTCGTGCGCTCT | To amplify a 1582-bp kanamycin cassette amplified from the vector pET30 |
| KM-R | TACCGTTCGTATAATGTATGCTATACGAAGTTATCAGGTGGCACTTTTCGGGGA |  |


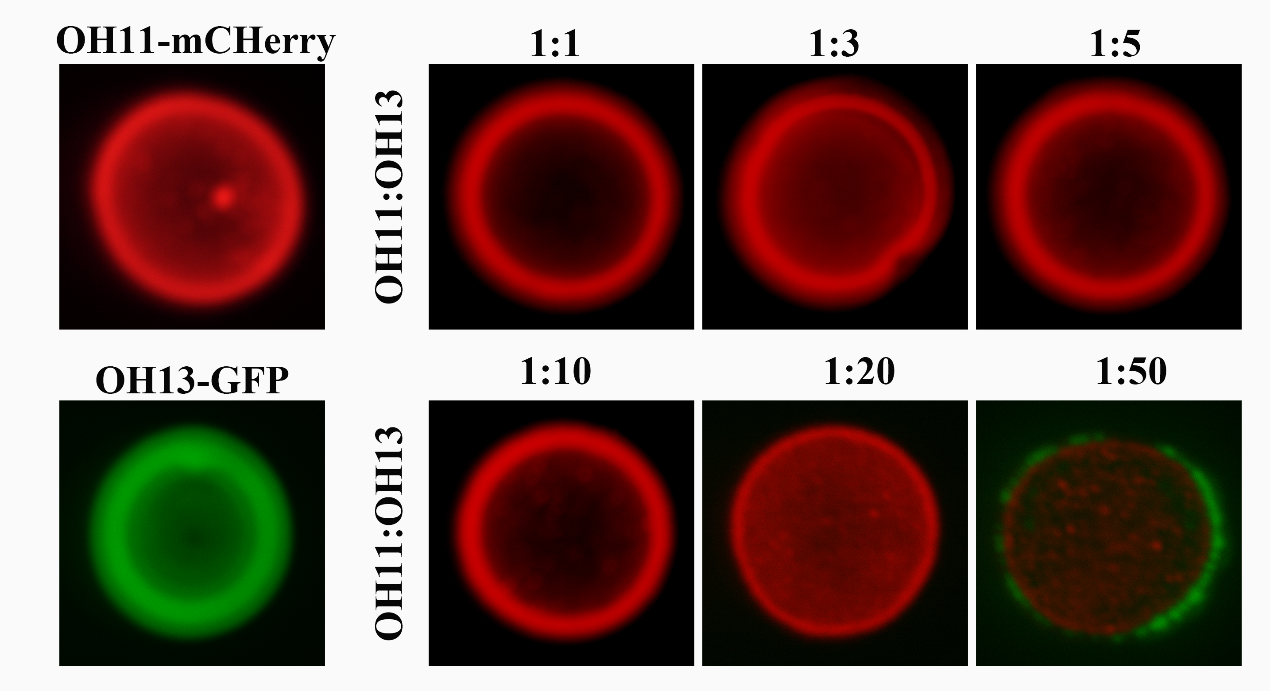
**Figure S1. Fluorescence evaluation of contact-dependent antibacterial activity by co-cultivating *L. enzymogenes* OH11 and *L. antibioticus* OH13 on 1/10 TSA agar at various ratios**. The fluorescence signals were observed after 24 hours of incubation. Wild-type OH11 and OH13 were labelled by mCherry and GFP, respectively. The selected co-cultivation ratios are shown.

**
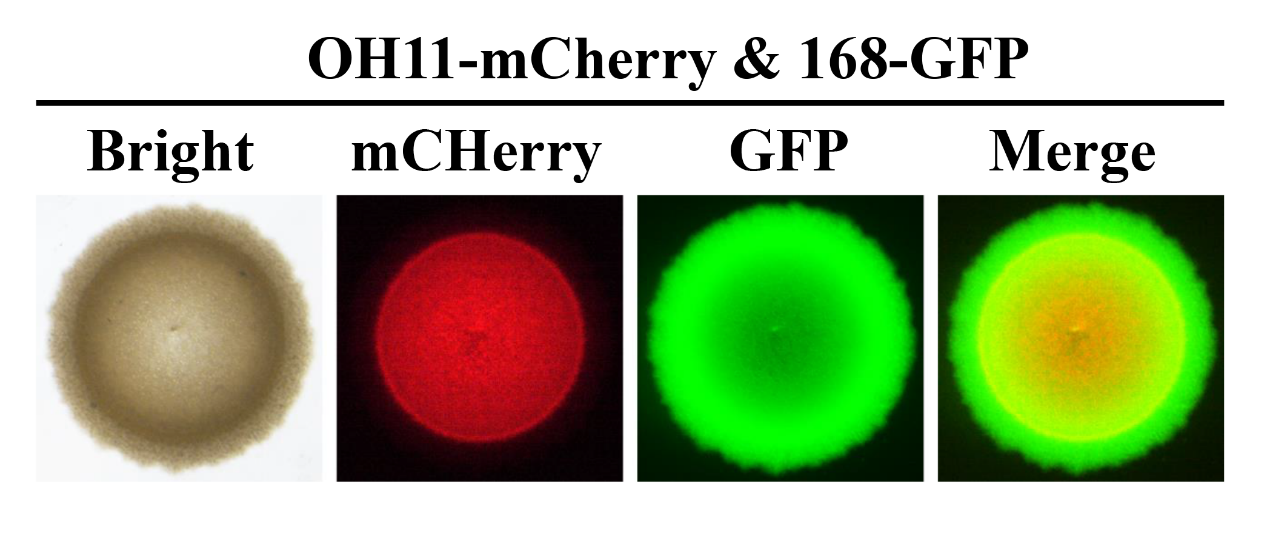
**

**Figure S2. Compatible cell-cell interaction between *L. enzymogenes* OH11 and *Bacillus subtilis* 168.** Wild-type OH11 and 168 were labelled by mCherry and GFP, respectively. The cultures of both strains were mixed at a ratio of 1:1 and co-incubated on 1/10 TSA agar. The fluorescence signals were observed after 24 hours of incubation.

**References**

Dong L, Guo Q, Wang P et al. (2020) Qualitative and quantitative analyses of the colonization characteristics of *Bacillus subtilis* strain NCD-2 on cotton root. Curr Microbiol **77**: 1600-1609 https://doi.org/10.1007/s00284-020-01971-y

Ling J, Wang H, Wu P et al. (2016) Plant nodulation inducers enhance horizontal gene transfer of *Azorhizobium caulinodans* symbiosis island. Proc Natl Acad Sci U S A **113**: 13875-13880. https://doi.org/10.1073/pnas.1615121113

Qian G, Hu B, Jiang Y, Liu F. (2009) Identification and Characterization of *Lysobacter enzymogenes* as a biological control agent against some fungal pathogens. Agricultural sciences in China. **8**: 68-75. https://doi.org/10.1016/S1671-2927(09)60010-9

Quandt J and Hynes M (1993) Versatile suicide vectors which allow direct selection for gene replacement in gram-negative bacteria. Gene 127: 15. https://doi.org/10.1016/0378-1119(93)90611-6

Shen X, Wang B, Yang N, et al. (2021). *Lysobacter enzymogenes* antagonizes soilborne bacteria using the type IV secretion system. Environ Microbiol **23**: 4673-4688. https://doi.org/10.1111/1462-2920.15662

Wang C, Pu T, Lou W et al. (2081) Hfq, a RNA chaperone, contributes to virulence by regulating plant cell wall-degrading enzyme production, type VI secretion system expression, bacterial competition, and suppressing host defense response in *Pectobacterium carotovorum*. Mol. Plant Microbe In 31: 1166-1178. https://doi.org/10.1094/MPMI-12-17-0303-R

Wang P, Chen H, Qian G, Liu, F (2017) LetR is a TetR family transcription factor from *Lysobacter* controlling antifungal antibiotic biosynthesis. Appl Microbiol Biot **101**: 3273-3282. https://doi.org/10.1007/s00253-017-8117-8

Yang M, Ren S, Shen D et al. (2020). An intrinsic mechanism for coordinated production of the contact-dependent and contact-independent weapon systems in a soil bacterium. PLoS Pathog 16: e1008967-e1008967. https://doi.org/10.1371/journal.ppat.1008967

Yin Z, Liu H, Li Z et al. (2015) Genome sequence of *Valsa* canker pathogens uncovers a potential adaptation of colonization of woody bark. The New Phytologist **208**: 1202-1216. https://doi.org/10.1111/nph.13544

Zhao Y, Qian G, Ye Y et al. (2016) Heterocyclic aromatic N-oxidation in the biosynthesis of phenazine antibiotics from *Lysobacter antibioticus*. Org Lett **18**: 2495-2498. https://doi.org/10.1021/acs.orglett.6b01089
